# Impacts of carbon nanomaterials on the diversity of microarthropods in turfgrass soil

**DOI:** 10.1038/s41598-017-01920-z

**Published:** 2017-05-11

**Authors:** Xue Bai, Shulan Zhao, Lian Duo

**Affiliations:** Tianjin Key Laboratory of Animal and Plant Resistance, College of Life Sciences, Tianjin Normal University, Tianjin, 300387 P.R. China

## Abstract

Nanoscale materials have been produced with unprecedented speed due to their widespread use, and they may eventually be released into the environment. As effective adsorbents for heavy metals, carbon nanomaterials can be used to immobilize metals in contaminated soil, but little information is available regarding their effects on soil microarthropods. This study was designed to investigate the influence of three types of carbon nanomaterials, graphene (G), graphene oxide (GO) and carbon nanotubes (CNTs) on soil microarthropod communities under turfgrass growth conditions. The application of carbon nanomaterials resulted in increased abundance of all soil microarthropods, especially in the GO and CNT treatments. GO also significantly increased the abundances of multiple trophic functional groups, including predators, detritivores, herbivores and fungivores. Further, the dominant genera varied among the treatments. Herbivorous microarthropods predominated in the control, whereas predatory species predominated in the carbon nanomaterial treatments. Carbon nanomaterials also increased the total taxonomic richness, Shannon diversity index, and dominance index of the microarthropod community, but they decreased the evenness index. Higher diversity of soil microarthropods indicates an environment suitable for soil mesofauna and for enhanced decomposition and nutrient cycling in the soil food web.

## Introduction

Soil microarthropods represent a class of soil fauna that is widespread in the soil ecosystem. These organisms play important roles in soil organic matter decomposition, nutrient mineralization, microbial activity and soil aggregation^1–3^, and are sensitive to the amendment of soil C and N and to disturbance of soil structure^4, 5^. Therefore, these organisms are good indicators reflecting the changes in ecosystems^4^. Changes in the community structure of microarthropods can be used to characterize soil conditions, such as the soil organic matter content, soil disturbances and pollution levels^4, 6^. Microarthropods include the Collembola (springtails), Acari (mites), Diptera and Coleoptera. In particular, Collembola and Acari are the most important and abundant microarthropod fauna in soils, and are the most valuable groups for soil quality evaluations^7^.

Carbon nanomaterials are composed of approximately 97–99% of carbon atoms in the form nearly spherical particles with diameters between 10 and 100 nm^8^. Oxidized carbon nanomaterials include a large amount of chemically bonded oxygen on the surface. Due to their large specific surface areas and porous structures with many functional groups, carbon nanomaterials have shown good adsorption abilities for metal ions in environmental applications^9, 10^. Tan *et al*.^11^ used graphene oxide (GO) membranes as adsorbents for removing heavy metals from water and found that the maximum adsorption capacities of the GO membranes for Cu^2+^, Cd^2+^ and Ni^2+^ were approximately 72.6, 83.8 and 62.3 mg/g, respectively, whereas Lu and Chiu^12^ used single-walled and multi-walled oxidized carbon nanotubes to adsorb Zn^2+^ in water and found that their adsorption capacities at pH = 7 and 25 °C were 43.66 and 32.68 mg/g, respectively. Adsorbents based on graphene are employed for removing pollutants from the environment^13^. Carbon nanomaterials, however, will inevitably be released into the soil environment after application, with mostly unknown consequences. Previous studies have evaluated the toxicological effects and the potential mechanisms of toxicity of carbon-based nanomaterials in bacteria, mammalian cells, and animal models^14, 15^, but very little work has explored the impacts of carbon nanomaterials on soil fauna, especially on the community of soil microarthropods.

The present study aimed to determine the impacts of three different carbon nanomaterials, graphene (G), graphene oxide (GO) and carbon nanotubes (CNTs) on the composition and diversity of the soil microarthropod community in a turfgrass system. We characterized the responses of soil microarthropods to carbon nanomaterials in order to provide a scientific basis for the application of carbon nanomaterials for remediating soil contaminated with heavy metals.

## Results

### The composition and abundance of soil microarthropods

In total, 24 genera of Acari and 8 genera of Collembola were recorded in this study (Table 1). The identified mites belonged to three suborders (Oribatida, Prostigmata, and Mesostigmata). The application of carbon nanomaterials significantly increased the species richness of soil microarthropods. Only 6 genera of soil microarthropods were found in the control soil, whereas 17, 18 and 11 genera were observed in G-, CNT- and GO-treated soils, respectively. The dominant genera varied among the treatments. The relative abundance of each genus in the control was greater than 10%. The dominant genera in the G treatments were *Palaeacarus* (Oribatida), *Spinibdella* (Prostigmata) and genera belong to Tarsonemidae (Prostigmata), accounting for 47.1% of the individuals. In the GO treatments, *Mahunkania* and *Cheylostigmaeus*, belonging to Prostigmata, were the dominant genera and presented 17.6% and 34.3%, respectively, of the total individuals. In the CNT treatments, there were four dominant taxa that together accounted for 76.5% of the individuals: *Brennandania* 28.1%, *Petalomium* 15.6%, Trombidiidae 17.2% and *Rhodacarellus* 15.6%.

**Table 1:** Composition of soil microarthropods in the different treatments (relative abundance % given in parentheses).

Taxon	Feeding	Control	Graphene	Graphene oxide	Carbon nanotube	References
Collembola		0.67 ± 0.33(22.3)^b^	2.33 ± 0.33(13.2)^b^	12.33 ± 1.20(18.1)^a^	0.67 ± 0.67(3.1)^b^	33
*Sinella*	Fun	0.00^b^	0.67 ± 0.33(3.8)^a^	0.00^b^	0.00^b^	
*Entomobrya*	Fun	0.00	0.00	0.00	0.67 ± 0.67(3.1)	
*Dicranocentrus*	Fun	0.00^b^	0.00^b^	2.67 ± 0.33(3.9)^a^	0.00^b^	
*Onychiurus*	Fun	0.00^c^	1.00 ± 0.00(5.7)^b^	3.00 ± 0.58(4.4)^a^	0.00^c^	
*Tullbergia*	Fun	0.67 ± 0.33(22.2)	0.67 ± 0.67(3.8)	0.00	0.00	
*Coloburella*	Fun	0.00^b^	0.00^b^	4.67 ± 1.45(6.9)^a^	0.00^b^	
*Paranurophorus*	Fun	0.00	0.00	1.00 ± 0.00(1.5)	0.00	
*Friesea*	Fun	0.00	0.00	1.00 ± 0.00(1.5)	0.00	
Oribatida		0.00	4.67 ± 2.62(26.4)	3.00 ± 0.58(4.4)	2.67 ± 0.67(12.5)	35
*Palaeacarus*	Det	0.00	4.33 ± 2.85(24.5)	1.00 ± 0.00(1.5)	0.00	
*Hypovertex*	Det	0.00	0.33 ± 0.33(1.9)	0.00	1.33 ± 1.33(6.3)	
*Epidamaeus*	Det	0.00^b^	0.00^b^	2.00 ± 0.58(2.9)^a^	1.00 ± 0.58(4.7)^ab^	
*Tepracarus*	Det	0.00	0.00	0.00	0.33 ± 0.33(1.6)	
Prostigmata		1.67 ± 0.67(55.6)^b^	8.67 ± 2.33(49.1)^b^	47.00 ± 6.81(69.1)^a^	13.33 ± 5.55(62.5)^b^	
*Bdella*	Pre	0.00	0.67 ± 0.67(3.8)	0.00	0.00	6, 19
*Spinibdella*	Pre	0.00	2.00 ± 1.15(11.3)	1.67 ± 0.33(2.5)	0.00	6, 19
*Pygmephorus*	Her	0.00	0.00	0.33 ± 0.33(0.5)	0.00	6, 19, 36
*Petalomium*	Her	0.00^c^	0.33 ± 0.33(1.9)^bc^	4.67 ± 0.88(6.9)^a^	3.33 ± 1.67(15.6)^ab^	19
*Mahunkania*	Her	0.00^b^	1.33 ± 0.88(7.5)^b^	12.00 ± 2.31(17.6)^a^	0.00^b^	19
*Brennandania*	Pre	0.00^b^	0.00^b^	4.67 ± 1.45(6.9)^ab^	6.00 ± 3.06(28.1)^a^	36
Caeculidae	Pre	0.00	0.67 ± 0.67(3.8)	0.00	0.00	6
Penthaleidae	Her	1.00 ± 0.58(33.3)	0.33 ± 0.33(1.9)	0.00	0.00	3
Trombidiidae	Pre	0.00^b^	0.00^b^	0.33 ± 0.33(0.5)^b^	3.67 ± 1.20(17.2)^a^	6
*Robustocheles*	Pre	0.00	0.67 ± 0.67(3.8)	0.00	0.00	34, 37, 38
*Neognathus*	Unknown	0.33 ± 0.33(11.1)	0.00	0.00	0.00	34, 38
Cheyletidae	Pre	0.00	0.67 ± 0.67(3.8)	0.00	0.00	6
*Cheylostigmaeus*	Pre	0.00^b^	0.00^b^	23.33 ± 8.82(34.3)^a^	0.33 ± 0.33(1.6)^b^	34
Tarsonemidae	Her	0.33 ± 0.33(11.1)^b^	2.00 ± 0.58(11.3)^a^	0.00^b^	0.00^b^	19
Mesostigmata		0.67 ± 0.33(22.3)^b^	2.00 ± 1.15(11.3)^ab^	5.67 ± 0.33(8.3)^a^	4.67 ± 2.33(21.9)^ab^	
Parasitidae	Pre	0.33 ± 0.33(11.1)	0.00	0.33 ± 0.33(0.5)	0.00	6
Phytoseiidae	Pre	0.33 ± 0.33(11.1)	0.00	0.00	0.33 ± 0.33(1.6)	19
*Kleemannia*	Pre	0.00^b^	0.00^b^	2.67 ± 0.33(3.9)^a^	1.00 ± 1.00(4.7)^ab^	33
*Gamasolaelaps*	Pre	0.00^b^	0.33 ± 0.33(1.9)^ab^	1.00 ± 0.00(1.5)^a^	0.00^b^	6
*Geholaspis*	Pre	0.00	1.67 ± 1.20(9.4)	1.67 ± 0.33(2.5)	0.00	6, 19
*Rhodacarellus*	Pre	0.00	0.00	0.00	3.33 ± 2.03(15.6)	19, 33
Total		3.00 ± 0.58^c^	17.67 ± 4.98^bc^	68.00 ± 7.00^a^	21.33 ± 4.81^b^	

Fun, fungivores; Det, detritivores; Pre, predators; Her, herbivores. Different letters indicate statistically significant differences between treatments, according to the LSD multiple range test (*p* < 0.05).

The abundance of total microarthropods was strongly affected by the treatments (Table 1). The average abundance of total soil microarthropods for the GO, CNT and G treatments was 22.7, 7.1 and 5.9 times higher, respectively, compared to the control treatment. However, no significant differences were observed between the G treatment and the control. The highest total abundance of soil microarthropods was observed with GO treatment, in which it was significantly higher than the soil microarthropod abundances in the G and CNT treatments. Prostigmata, with the highest abundance, accounted for 55.6%, 49.1%, 69.1%, and 62.5% of the total soil microarthropods in the control, G, GO, and CNT treatments, respectively. Compared with the control and the other two treatments, GO significantly increased the abundances of Prostigmata and Collembola.

### Soil microarthropod trophic groups

**Figure 1:**
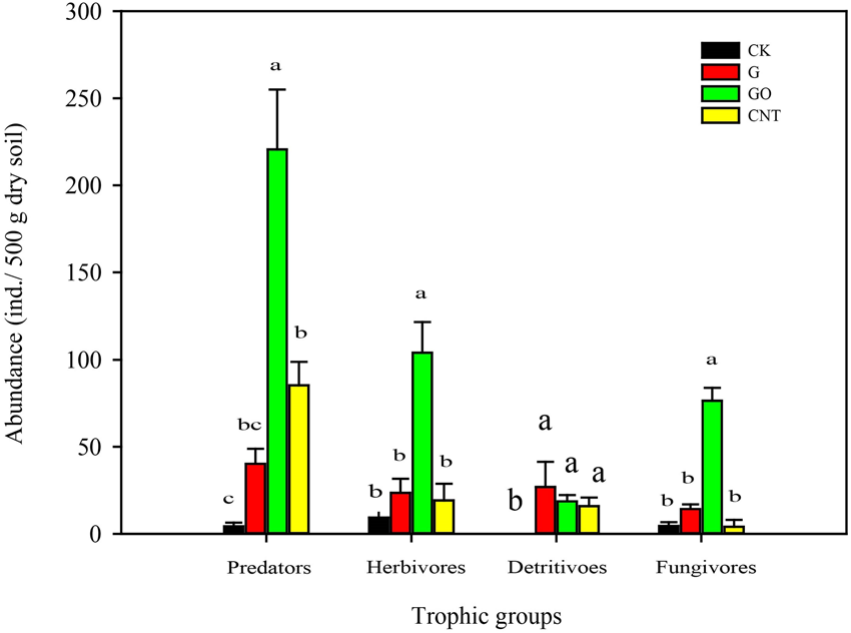
Abundances of four trophic groups of soil microarthropods in the different treatments. Bars represent standard deviation with the mean. Different letters indicate statistically significant differences between treatments, according to the LSD multiple range test (*p* < 0.05).

All of the collected soil microarthropods except for *Neognathus* were grouped into fungivorous, detritivorous, herbivorous and predatory guilds according to their feeding habits and the results for their abundances are shown in Fig. 1. The carbon nanomaterials increased the total abundance of each trophic group. Detritivorous microarthropods were not found in the control but were present in all of the carbon nanomaterial treatments. The GO treatment showed the most significant effects, and the abundances of predators, herbivores, and fungivores were increased by 51-fold, 10-fold and 16-fold, respectively, compared to the control. Herbivorous microarthropods dominated in the control, accounting for 44.4% of the total individuals, whereas predators dominated in the G, GO and CNT treatments, representing 37.8%, 52.6% and 68.8%, respectively, of all individuals (Supplementary Dataset ﻿1).

### Soil microarthropod community diversity

The diversity indices were significantly influenced by all the carbon nanomaterials (Table 2). Significant increases (*P* < 0.05) in the Shannon–Wiener index and dominance relative to control values were observed in all of the carbon nanomaterial treatments. Moreover, the GO and G treatments showed significantly greater species richness than did the CNT and control treatments. Although the GO treatment showed lower evenness, the evenness indices were not significantly different among the G, CNT and control treatments.

**Table 2:** Diversity indices of the soil microarthropod community under the different treatments.

Treatments	Shannon index (*H*′)	Richness index (*SR*)	Evenness index (*J*)	Dominance index (*C*)
Control	0.94 ± 0.13^c^	1.57 ± 0.13^c^	0.98 ± 0.02^a^	0.60 ± 0.05^b^
Graphene	1.98 ± 0.02^a^	2.77 ± 0.15^b^	0.92 ± 0.04^a^	0.84 ± 0.02^a^
Graphene oxide	2.17 ± 0.14^a^	3.57 ± 0.19^a^	0.78 ± 0.05^b^	0.81 ± 0.04^a^
Carbon nanotube	1.55 ± 0.07^b^	1.56 ± 0.03^c^	0.90 ± 0.05^ab^	0.76 ± 0.02^a^

Different letters within the same column indicate statistically significant differences between treatments, according to the LSD multiple range test (*p* < 0.05).

## Discussion

Soil microarthropods are important components of the soil ecosystem. The species composition and abundance of soil microarthropods, the trophic functional groups, and the community indices can be used as sensitive indicators for evaluating ecosystem processes^4^. Organic fertilizers may promote increases in faunal populations by increasing the quality and quantity of food needed by soil microarthropods^5, 16, 17^. Greater microarthropod densities with increased soil fertility were also observed by Cole *et al*.^18^. However, Cao *et al*.^19^ found that applications of chemical fertilizer decreased the number of soil mites, perhaps because of the direct toxicity of metal contaminants in the chemical fertilizers^20^. In the present study, the abundance of soil microarthropods significantly increased in the turfgrass plantation under the application of G, GO and CNT. This may be attributable to an increase in the supply of soil nutrients (i.e., soil C) through the addition of carbon nanomaterials. Previous studies demonstrated that the growth of soil microarthropods abundance depends on increases in the soil C and N concentrations^5^.

Both graphene and graphene oxide increased the diversity and richness of soil microarthropods, effects that were mainly attributable to the marked increases in Oribatida and Prostigmata. In a previous study, an increase in the diversity of soil fauna was correlated to an increase in the diversity of available food resources^21^. In general, application of a low level of fertilizer tends to result in increased faunal diversity due to its positive effect on detrital inputs^5, 17^. However, graphene oxide showed the most significant effect on the abundance and diversity of soil microarthropods. Previous studies demonstrated that GO can enhance cell adhesion and proliferation and promote biological growth and reproduction, thereby affecting the populations and diversity of total microarthropods^22^.

The function of soil and the maintenance of soil quality are closely linked to the micro-fauna community composition; therefore, understanding the responses of soil microarthropod functional groups is useful for revealing the ecological effects of carbon nanomaterials^23^. Based on the presence of *Oribatida* in soils treated with carbon nanomaterials, these soils tend to contain nearly all functional groups of microarthropods^24^. In the present study, *Oribatida* was mainly represented by detritivores, which are sensitive to the soil environments^25^. The greater abundances of *Oribatida* in carbon nanomaterial treatments suggest that the nutrient level and soil quality were improved. Furthermore, detritivorous *Oribatida* are involved in organic matter decomposition, nutrient cycling and soil formation in the soil ecosystem^1, 25^. In the GO treatment, the number of microarthropods in predatory, herbivorous and fungivorous groups increased at a significance level of 51-fold, 10-fold and 16-fold, respectively, in comparison with the control, whereas no significant differences in abundance of these trophic groups were found among the control, G and CNT treatments. This result indicates that different trophic communities have different adaptive mechanisms to the treatments. Predatory microarthropods were found to be dominant in carbon nanomaterial-treated soils, whereas herbivorous species were dominant in the control treatment. The increase in predatory abundance was likely caused by an increase in the availability of prey species, e.g., Enchytraeidae and nematodes^26^. Nematodes are a preferred food source for most predatory species^27, 28^. As suggested in previous studies, predators may more directly regulate the abundances of detritivorous and fungivorous microarthropods by limiting their population expansion in the natural soil^29, 30^. Further studies should be conducted to identify the mechanisms by which carbon nanomaterials affect soil microarthropods, perhaps through effects on the soil properties.

In conclusion, the application of carbon nanomaterials (G, GO and CNT) in turfgrass soil significantly increased the abundance and diversity of soil microarthropods. The GO treatment produced significant increases in the abundances of four trophic microarthropods. Predatory arthropods predominated in the carbon nanomaterial treatments, whereas herbivorous species predominated in the control. The increased microarthropod populations support enhanced decomposition and nutrient cycling in the soil food web by strengthening both bottom-up and top-down processes.

## Materials and Methods

### Experimental design

The tested soil was gathered from the top 20 cm of an experimental site at the Tianjin Normal University campus. It is a sandy loam with a pH (in water) of 7.3, water content of 19.4%, conductivity of 2250 μS cm^−1^, and organic matter, total phosphorus, and total nitrogen contents of 52.3, 3.75 and 2.15 g kg^−1^, respectively. *Festuca arundinacea* Schreb. was selected as the tested turfgrass. Municipal solid waste (MSW) compost was obtained from Xiaodian composting plants in Tianjin, China. The properties and origins of the carbon nanomaterials, graphene (G), graphene oxide (GO) and carbon nanotubes (CNTs), are reported in Table 3.

**Table 3:** The properties and origins of the carbon nanomaterials used in this study.

Carbon nanomateials	Origin	Shape	Size	Specific surface area (m^2^ g^−1^)
Graphene	JCNANO Technology Co. Ltd., Nanjing, China	Black flake	0.5–20 μm	40–60
Graphene oxide	Hengqiu Graphene Nanotechnology Co. Ltd., Suzhou, China	Black or brown yellow powder	Thickness 3.4–7.0 nm; diameter 10–50 μm	100–300
Carbon nanotube	Boyu Gaoke new material technology Co. Ltd., Beijing, China	Black powder	Diameter 20–40 nm; length 10–30 μm	>110

For this study 1500 g of tested soil, 50 g of MSW compost and 15 g of carbon nanomaterials were mixed thoroughly and transferred to plastic pots (height 15 cm; inner diameter 20 cm). Treatments without the nanomaterials were used as the controls, and each treatment was replicated three times. Then, 5 g of seeds of *Festuca arundinacea* was sown in each pot. Cultivation was performed in a greenhouse under natural light conditions (646-27090 LX). During the experiment, the night and day average temperatures were 15 °C and 26 °C, respectively, and the relative humidity was between 35% and 55%. Water was supplied daily to maintain adequate substrate moisture for turfgrass growth. Turfgrasses were mowed once, on day 65.

### Characterization of soil microarthropods

Soil samples were taken from the experimental pots on day 130. Soil microarthropods were extracted from 100 g of fresh soil using the Tullgren method^31^ and then preserved in 70% alcohol. All extracted faunal samples were sorted and counted under a dissection microscope and identified to the genus level^32^. The soil microarthropods were grouped into predatory, herbivorous, detritivorous and fungivorous guilds^3, 6, 19, 33–38^.

### Statistical analysis

The Shannon-Wiener index (*H*′), Margalef richness index (*SR*), Pielou evenness index (*J*) and Simpson dominance index (*C*) were used to describe the diversity of the soil microarthropod community^39^. *H*′ *=* −∑*Pi*ln*Pi*; *SR* = (*S* − 1)/ln*N*; *J* = *H*′/ln*S*; *C* = 1 − ∑(*Pi*)^2^, where *S* is the number of species, *N* is the total number of individuals and *Pi* = *Ni*/*N* is the ratio between the individual number in a genus and the total number of individuals.

The responses of the microarthropod community structure to carbon nanomaterials were examined by using analysis of variance (ANOVA). Differences between means were evaluated by using the least significant difference (LSD) test. All statistical tests were conducted at a significance level of *p* < 0.05 using the SPSS 17.0 software package (SPSS Inc., Chicago, USA). Data presented in the Tables and Figure are means ± standard deviations (SD) of three replicates for each treatment. SigmaPlot 12.5 was used to plot the graph^40^.

## Electronic supplementary material

Dataset 1
