# Supplementary material for: Impacts of carbon nanomaterials on the diversity of microarthropods in turfgrass soil
Source: Sci Rep. 2017 May 11;7:1779. doi: 10.1038/s41598-017-01920-z (PMC5431980; doi:10.1038/s41598-017-01920-z)
Supplement: Supplementary file 1 — Dataset 1 [file 41598_2017_1920_MOESM1_ESM.doc]

**Impacts of carbon nanomaterials on the diversity of microarthropods in turfgrass soil[[1]](#footnote-2)***

**Xue Bai^1^, Shulan Zhao^1^ & Lian Duo^1^**

**Abundances (ind./500 g dry soil) of four trophic soil microarthropods in the different treatments for figure 1**

| **Treatments** | **Predators** | **Herbivores** | **Detritivores** | **Fungivores** |
| --- | --- | --- | --- | --- |
| **Control** | 4.27±2.16^c^ | 9.23±2.85^b^ | 0.00±0.00^b^ | 4.41±2.3^b^ |
| **G** | 40.20±8.63^bc^ | 23.5±8.1^b^ | 26.9±12.5^a^ | 14.2±2.7^b^ |
| **GO** | 220±34^a^ | 104±17^a^ | 18.6±3.7^a^ | 76.5±7.4^a^ |
| **CNT** | 85.3±13.5^b^ | 19.2±9.5^b^ | 15.8±5.0^a^ | 4.0±4.0^b^ |

Different letters indicate statistically significant differences between treatments, according to the LSD multiple range test (*p* < 0.05).

1. ^1^Tianjin Key Laboratory of Animal and Plant Resistance, College of Life Sciences, Tianjin Normal University, Tianjin 300387, P.R. China. Correspondence and requests for materials should be addressed to L.D.(email: [duolian_tjnu@163.com](mailto:duolian_tjnu@163.com)) or S.Z.(email: [zhaosl_tjnu@126.com](mailto:zhaosl_tjnu@126.com)) [↑](#footnote-ref-2)
